# Supplementary material for: Phytochemical Screening, Antibacterial, Antifungal, Antiviral, Cytotoxic, and Anti-Quorum-Sensing Properties of Teucrium polium L. Aerial Parts Methanolic Extract
Source: Plants (Basel). 2020 Oct 23;9(11):1418. doi: 10.3390/plants9111418 (PMC7690738; doi:10.3390/plants9111418)

Supplementary Information (S1)

10-Hydroxyloganin:

Compound Label	Name	*m/z*	RT	Algorithm	Mass
Cpd 2: 10-Hydroxyloganin; C17 H26 O11; 0.955 89.0288	**10-Hydroxyloganin**	387.1261	0.955	Auto MS/MS	406.1439

MS Spectrum

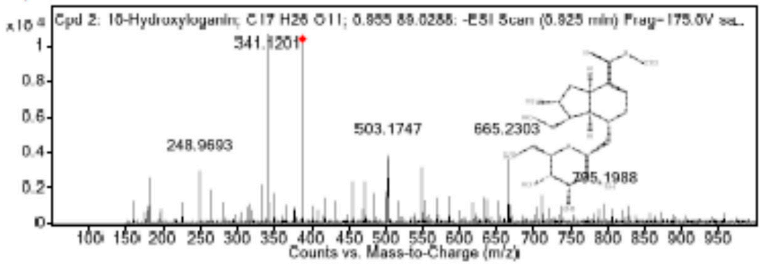

MS Zoomed Spectrum

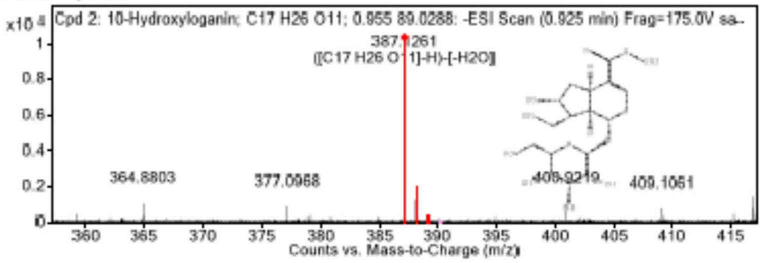

MS Spectrum Peak List

m/z	*Calc m/z*	Diff(ppm)	z	Abund	Formula	Ion
181.0785				2558.75		
248.9693				2987.06		
341.1201			1	11562.77		
387.1261	387.1297	9.15	1	10678.21	C17 H26 O11	(M-H)-[-H2O]
388.129	388.1331	10.61	1	1322.19	C17 H26 O11	(M-H)-[-H2O]
389.1313	389.1351	9.68	1	352.2	C17 H26 O11	(M-H)-[-H2O]
471.0878				2476.53		
503.1747			1	3892.37		
549.1807			1	3389.31		
665.2303			1	3921.29		

MSMS Spectrum

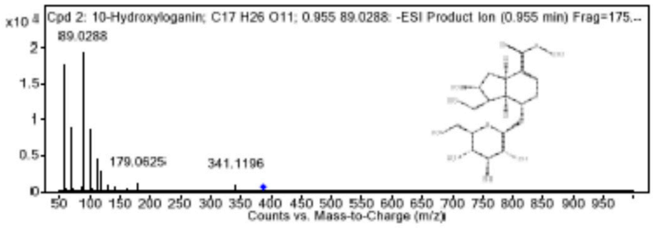

MS/MS Spectrum Peak List

m/z	z	Abund
59.017	1	17862.52
71.0171		9642.09
89.0288	1	19550.53
101.0289		8743.63
113.0294		4654.55
119.0397		3006.39
131.0397		931.97
143.0409		624.03
179.0625		1182.16
341.1196		877.45

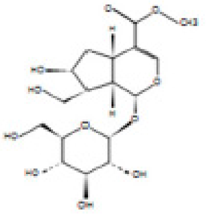

Compound Structure

13. R-hydroxy-9E,11Z octadecadienoic acid:

Compound Label	Name	*m/z*	RT	Algorithm	Mass
Cpd 2: 13R-hydroxy-9E,11Z-octadecadienoic acid; C18 H32 O3; 1.046 158.1156	**13R-hydroxy-9E,11Z-octadecadienoic acid**	301.2106	1.046	Auto MS/MS	296.232

MS Spectrum

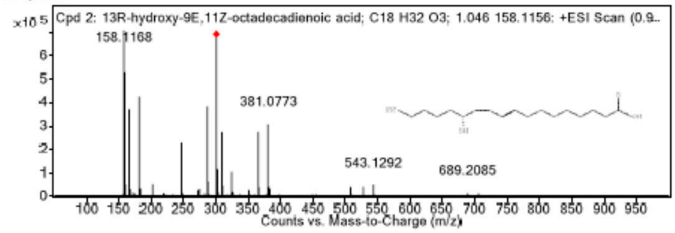

MS Zoomed Spectrum

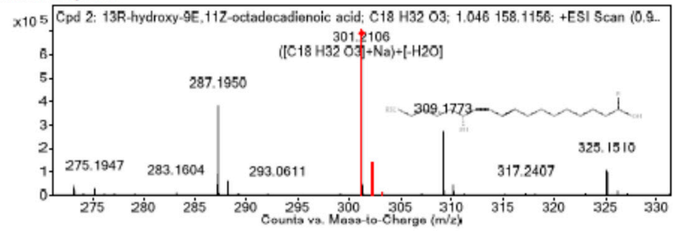

MS Spectrum Peak List

m/z	*Calc m/z*	Diff(ppm)	z	Abund	Formula	Ion
158.1168			1	5673944.95		
159.1198			1	533659.77		
166.0825			1	381549.47		
182.0565			1	422181.12		
287.195			1	407742.18		
301.2106	301.2138	10.47	1	708625.21	C18 H32 O3	(M+Na)+[-H2O]
302.2138	302.2172	11.13	1	116183.51	C18 H32 O3	(M+Na)+[-H2O]
303.2181	303.2202	6.96	1	15033.02	C18 H32 O3	(M+Na)+[-H2O]
365.1034			1	285862.7		
381.0773			1	313796.47		

MSMS Spectrum

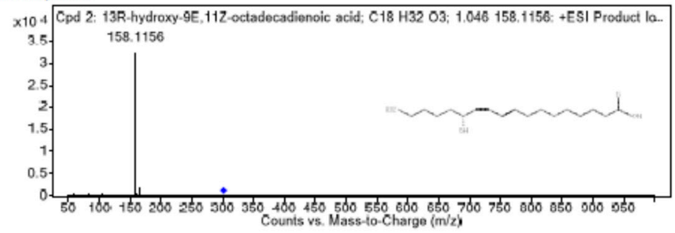

MS/MS Spectrum Peak List

m/z	z	Abund
58.0639		513.53
84.0798		402.7
98.0966		70.22
104.1055		245.49
121.0341		82.29
158.1156	1	33056.74
158.7403		84.84
159.1189	1	2775.32
160.1219	1	278.51
166.0815		1470.26

Compound Structure

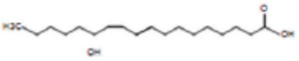

Bis (2-hydroxypropyl) amine:

Compound Label	Name	*m/z*	RT	Algorithm	Mass
Cpd 3: Bis (2-hydroxypropyl) amine; C6 H15 N O2; 1.062	**Bis (2-hydroxypropyl) amine**	156.1004	1.062	Auto MS/MS	133.1111

MS Spectrum

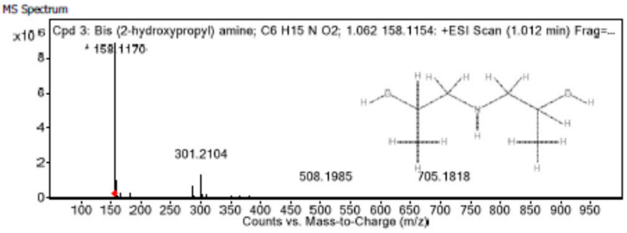

MS Zoomed Spectrum

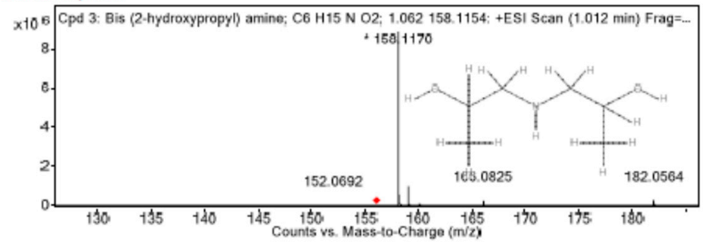

MS Spectrum Peak List

m/z	*Calc m/z*	Diff(ppm)	z	Abund	Formula	Ion
156.1004	156.0995	-5.81		11036.97	C6 H15 N O2	(M+Na)+
157.1021	157.1026	3.15	1	1002.12	C6 H15 N O2	(M+Na)+
158.117			1	9510118.55		
159.1198			1	952203.25		
166.0825			1	245746.76		
182.0564			1	251645.65		
287.1951			1	742533.83		
301.2104			1	1302384.32		
302.2136			1	203261.45		
309.1766			1	198192.2		

MSMS Spectrum

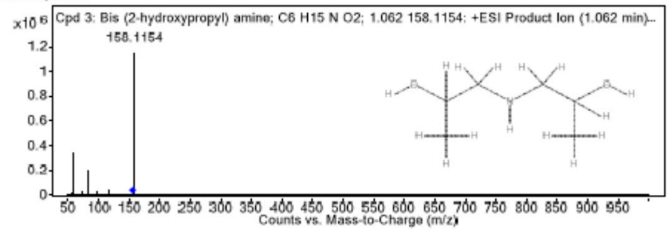

MS/MS Spectrum Peak List

m/z	z	Abund
55.0531		17065.35
58.0641	1	360634.6
71.0481		9938.97
72.0799		21690
73.0641		13618.47
84.0798		206056.16
98.0953		21891.98
116.0693		33556.68
158.1154	1	1171914.73
159.1188	1	59576.96

Compound Structure

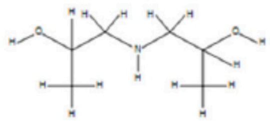

9. -amino-nonanoic acid:

Compound Label	Name	*m/z*	RT	Algorithm	Mas[illegible]
Cpd 5: 9-amino-nonanoic acid; C9 H19 N O2; 1.447 174.1469	9-amino-nonanoic acid	174.1474	1.447	Auto MS/MS	173.[illegible]

MS Spectrum

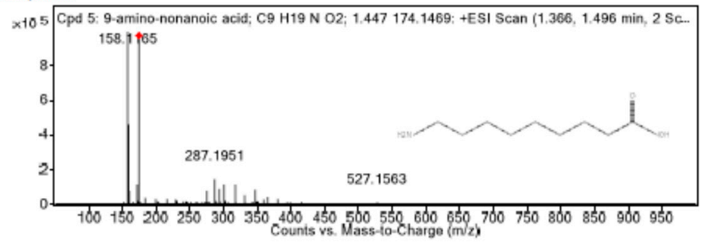

MS Zoomed Spectrum

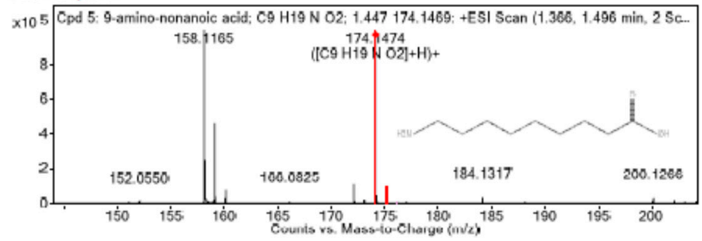

MS Spectrum Peak List

m/z	*Calc m/z*	Diff(ppm)	z	Abund	Formula	Ion
158.1165			1	5232643.96		
159.1197			1	466897.16		
172.1319			1	116016		
174.1474	174.1489	8.55	1	995937.32	C9 H19 N O2	(M+H)+
175.1508	175.1521	7.44	1	93048.62	C9 H19 N O2	(M+H)+
287.1951			1	154972.47		
294.1527			1	92522.72		
301.2104			1	112601.05		
317.2419			1	112301.71		
347.2882			1	83533.3		

MSMS Spectrum

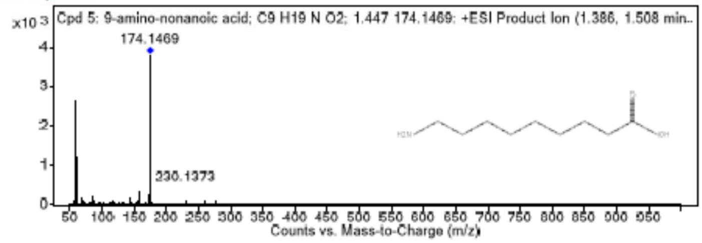

MS/MS Spectrum Peak List

m/z	z	Abund
58.0641		2807.36
59.0721		2170.02
60.0795		1288.34
69.0692		161.56
86.0945		195.6
144.0998		168.8
158.1145	1	287.9
172.1318		238.95
174.1469	1	3810.31
175.1488	1	235.91

Compound Structure

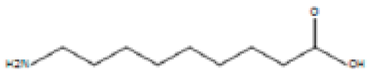

10-Amino-decanoic acid:

Compound Label	Name	*m/z*	RT	Algorithm	Mass
Cpd 11: 10-amino-decanoic acid; C10 H21 N O2; 3.807 188.1626	**10-amino-decanoic acid**	188.1633	3.807	Auto MS/MS	187.156

MS Spectrum

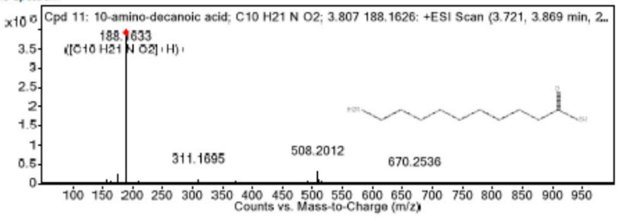

MS Zoomed Spectrum

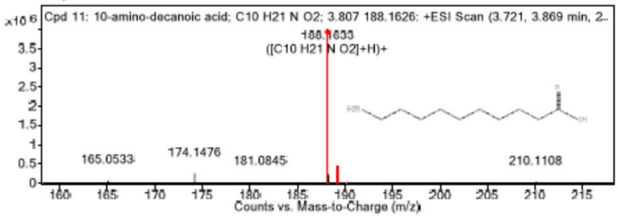

MS Spectrum Peak List

m/z	*Calc m/z*	Diff(ppm)	z	Abund	Formula	Ion
158.116			1	105679.29		
165.0533			1	69268.36		
174.1476			1	244009.31		
188.1633	188.1645	6.36	1	4016534.24	C10 H21 N O2	(M+H)+
189.1666	189.1677	6.19	1	405481.94	C10 H21 N O2	(M+H)+
210.1108			1	39420.94		
311.1695			1	97321.72		
491.1739			1	41001.01		
508.2012			1	313611.1		
509.2042			1	78059.34		

MSMS Spectrum

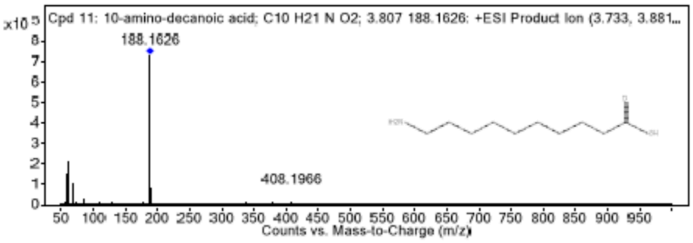

MS/MS Spectrum Peak List

m/z	z	Abund
58.0643		1334.93
59.0717		1630.28
60.0798	1	2194.79
61.0843	1	188.91
69.0696		989
73.0644		124.03
87.0434		273.47
129.0899		145.96
188.1626	1	7369.94
189.1651	1	856.37

Compound Structure

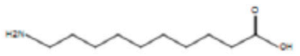

7-Epiloganin tetraacetate:

Compound Label	Name	*m/z*	RT	Algorithm	Mass
Cpd 6: 7-Epiloganin tetraacetate; C25 H34 O14; 4.739 353.0790	**7-Epiloganin tetraacetate**	593.1681	4.739	Auto MS/MS	558.1987

MS Spectrum

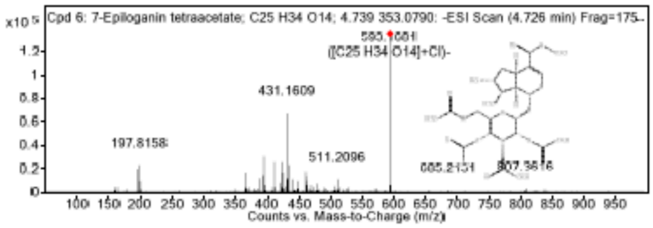

MS Zoomed Spectrum

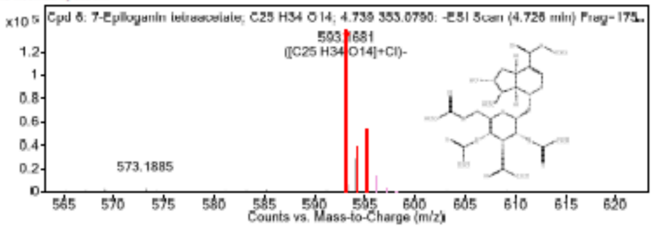

MS Spectrum Peak List

m/z	*Calc m/z*	Diff(ppm)	z	Abund	Formula	Ion
195.8185				19854.34		
197.8158				23092.49		
395.1835			1	31027.28		
411.1789			1	25558.41		
423.1917			1	25544.3		
431.1609			1	71623.88		
433.2208			1	23023.82		
593.1681	593.1643	-6.43	1	140483.21	C25 H34 O14	(M+Cl)-
594.1704	594.1677	-4.62	1	29701.54	C25 H34 O14	(M+Cl)-
595.1718	595.1628	-15.1	1	5743.22	C25 H34 O14	(M+Cl)-

MSMS Spectrum

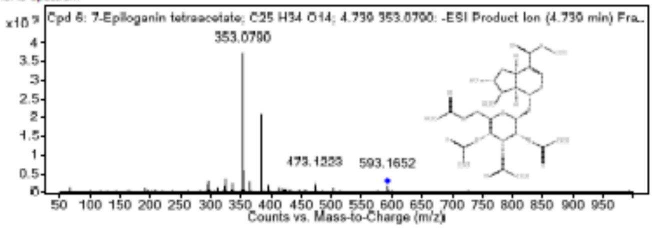

MS/MS Spectrum Peak List

m/z	z	Abund
296.0785		217.26
297.0874		333.98
325.0825		352.15
337.0823		246
353.079	1	3773.01
354.0807	1	595.91
365.0792		292.01
383.0895	1	2223.39
384.0918	1	413.59
473.1223	1	225.2

Compound Structure

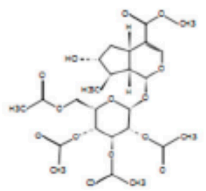

b-D-Glucopyranosideuronic acid, 6-(3-oxobutyl)-2- naphthalenyl:

Compound Label	Name	m/z	RT	Algorithm	Mass
Cpd 9: b-D-Glucopyranosiduronic acid, 6-(3-oxobutyl)-2-naphthalenyl; C20 H22 O8; 5.342 121.0344	**b-D-Glucopyranosiduronic acid, 6-(3-oxobutyl)-2-naphthalenyl**	371.1106	5.342	Auto MS/MS	390.1284

MS Spectrum

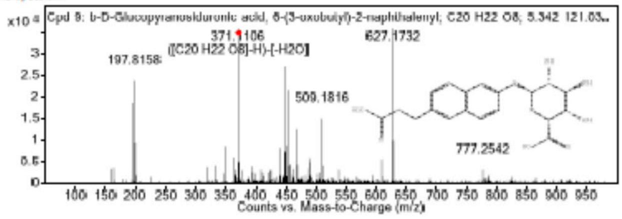

MS Zoomed Spectrum

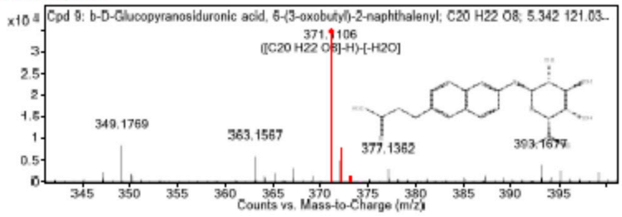

MS Spectrum Peak List

m/z	Calc m/z	Diff(ppm)	z	Abund	Formula	Ion
195.8185				18756.86		
197.8158				23703.31		
371.1106	371.1136	8.18	1	35925.37	C20 H22 O8	(M-H)-[-H2O]
372.1136	372.117	9.11	1	5110.07	C20 H22 O8	(M-H)-[-H2O]
373.1154	373.1194	10.85	1	1145.98	C20 H22 O8	(M-H)-[-H2O]
447.1569			1	27609.22		
453.1902			1	22115.58		
467.1697			1	12503.12		
509.1816			1	14925.38		
627.1732			1	39129.89		

MSMS Spectrum

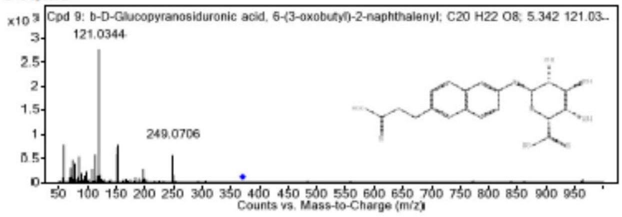

MS/MS Spectrum Peak List

m/z	z	Abund
59.0172		784.66
71.0168		306.48
75.0118		473.57
77.0431		408.44
85.0337		526.84
113.0301		575.13
121.0344	1	2805.62
152.0181		784.89
197.0523		263.19
249.0706		583.85

Compound Structure

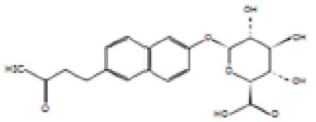

Cepharanthine:

Compound Label	Name	*m/z*	RT	Algorithm	Mass
Cpd 17: CEPHARANTHINE; C37 H38 N2 O6; 5.948	**CEPHARANTHINE**	623.2169	5.948	Auto MS/MS	606.2582

MS Spectrum

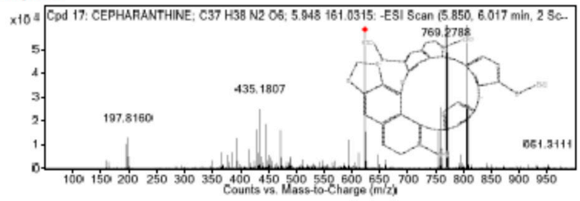

MS Zoomed Spectrum

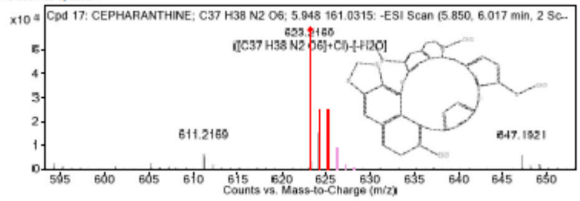

MS Spectrum Peak List

m/z	*Calc m/z*	Diff(ppm)	z	Abund	Formula	Ion
623.2169	623.2318	23.88	1	60267.22	C37 H38 N2 O6	(M+Cl)-[-H2O]
624.2196	624.2351	24.79	1	15537.01	C37 H38 N2 O6	(M+Cl)-[-H2O]
625.2221	625.231	14.13	1	3155.94	C37 H38 N2 O6	(M+Cl)-[-H2O]
759.1973			1	25832.75		
769.2788			1	599955.54		
770.2824			1	221005.34		
771.2839			1	50272.61		
805.2553			1	80261.79		
806.2579			1	25112.03		
807.254			1	26750.29		

MSMS Spectrum

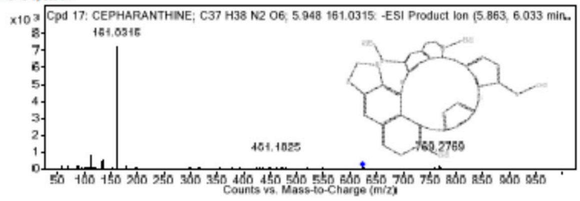

MS/MS Spectrum Peak List

m/z	z	Abund
59.0174		151.3
71.0179		194.72
85.0334		149.26
113.0297		843.36
133.0359		411.8
135.0514		503.02
161.0315	1	7250.46
162.0347	1	538.98
179.0422		148.09
769.2769		180.16

Compound Structure

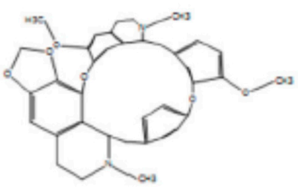

Rhoifolin:

Compound Label	Name	*m/z*	RT	Algorithm	Mass
Cpd 14: RHOIFOLIN; C27 H30 O14; 6.376 271.0587	**RHOIFOLIN**	579.1707	6.376	Auto MS/MS	578.1634

MS Spectrum

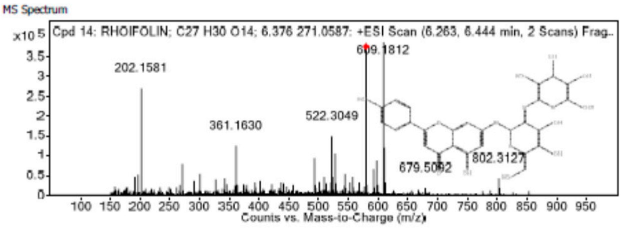

MS Zoomed Spectrum

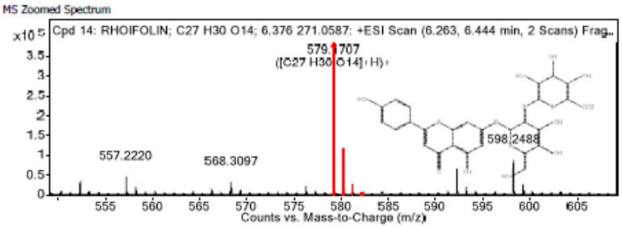

MS Spectrum Peak List

m/z	*Calc m/z*	Diff(ppm)	z	Abund	Formula	Ion
202.1581			1	269161.63		
361.163			1	126052.71		
522.3049			1	148761		
528.2081			1	107718.2		
579.1707	579.1708	0.14	1	388341.37	C27 H30 O14	(M+H)+
580.1737	580.1742	0.86	1	108400.34	C27 H30 O14	(M+H)+
581.1764	581.1766	0.33	1	25629.51	C27 H30 O14	(M+H)+
582.182	582.1793	-4.62	1	5744.97	C27 H30 O14	(M+H)+
609.1812			1	413623.55		
610.1844			1	122764.61		

MSMS Spectrum

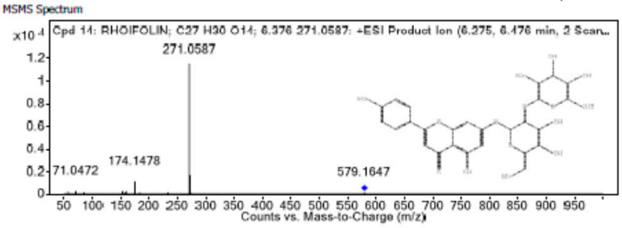

MS/MS Spectrum Peak List

m/z	z	Abund
57.033		209.52
71.0472		257.53
153.0149		247.2
160.1338		249.69
174.1478	1	1131.15
175.1496	1	155.17
271.0587	1	11571.27
272.0624	1	1750.59
273.0656	1	153.6
579.1647		153.9

Compound Structure

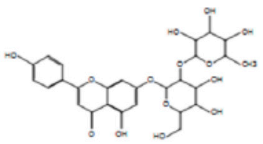

Sericetin diacetate:

Compound Label	Name	m/z	RT	Algorithm	Mass
Cpd 19: SERICETIN DIACETATE; C29 H28 O7; 7.838 373.1261	SERICETIN DIACETATE	493.1695	7.838	Auto MS/MS	488.191

MS Spectrum

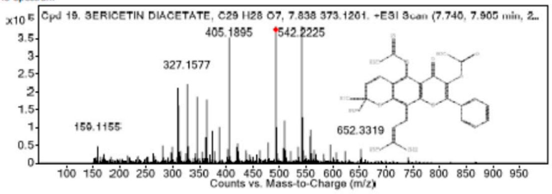

MS Zoomed Spectrum

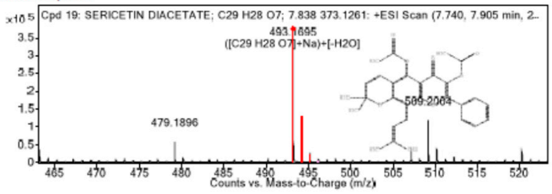

MS Spectrum Peak List

m/z	*Calc m/z*	Diff(ppm)	z	Abund	Formula	Ion
309.1474			1	214091.64		
311.1266			1	168544.2		
327.1577			1	234131.4		
345.1683			1	195699.9		
363.1789			1	177180.01		
405.1895			1	354902.91		
493.1695	493.1622	-14.81	1	399052.47	C29 H28 O7	(M+Na)+[-H2O]
494.1729	494.1655	-14.78	1	101394.07	C29 H28 O7	(M+Na)+[-H2O]
495.1798	495.1684	-22.97	1	27662.45	C29 H28 O7	(M+Na)+[-H2O]
542.2225			1	464357.12		

MSMS Spectrum

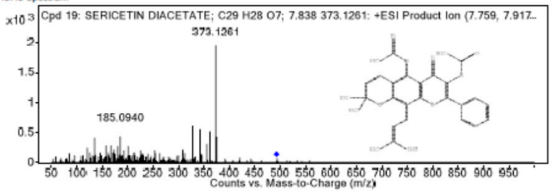

MS/MS Spectrum Peak List

m/z	z	Abund
135.0783		435.48
157.1002		284.92
167.0834		281.51
185.094		437.17
327.1197		628.18
343.1152		555.31
355.1158		307.26
361.1274		517.51
373.1261	1	2026.64
374.1287	1	443.42

Compound Structure

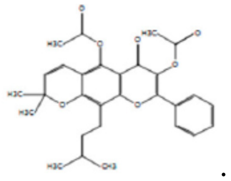

Troxerutin:

Compound Label	Name	m/z	RT	Algorithm	Mass
Cpd 18: TROXERUTIN; C33 H42 O19; 5.960 285.0503	**TROXERUTIN**	759.1965	5.96	Auto MS/MS	742.2379

MS Spectrum

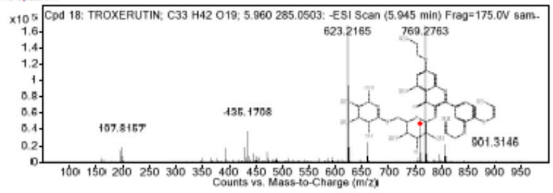

MS Zoomed Spectrum

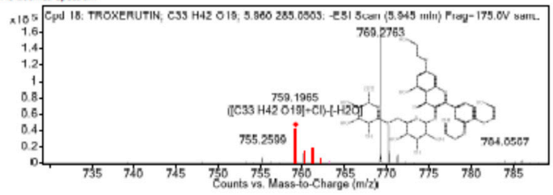

MS Spectrum Peak List

m/z	Calc m/z	Diff(ppm)	z	Abund	Formula	Ion
435.1798			1	37654.29		
623.2165			1	351469.87		
624.219			1	94129.03		
659.1923			1	25041.53		
759.1965	759.1909	-7.38	1	42544.08	C33 H42 O19	(M+Cl)-[-H2O]
760.1991	760.1943	-6.4	1	12630.28	C33 H42 O19	(M+Cl)-[-H2O]
761.2015	761.1901	-14.99	1	2865.43	C33 H42 O19	(M+Cl)-[-H2O]
762.2051	762.1926	-16.49	1	832.85	C33 H42 O19	(M+Cl)-[-H2O]
769.2763			1	167313.4		
770.2789			1	49290.49		

MSMS Spectrum

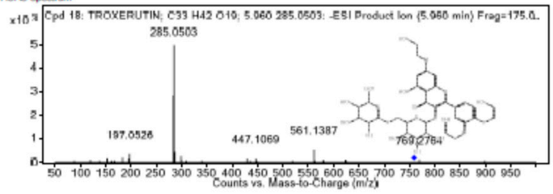

MS/MS Spectrum Peak List

m/z	z	Abund
153.061		180.53
183.0378		195.11
197.0526		346.54
284.0424		3076.31
285.0503	1	4986
286.0529	1	492.76
299.0638	1	286.14
447.1069		160.08
561.1387	1	543.91
562.1441	1	289.7

Compound Structure

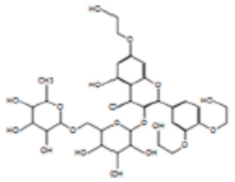

Deoxyloganin tetraacetate:

Compound Label	Name	*m/z*	RT	Algorithm	Mass
Cpd 23: Deoxyloganin tetraacetate; C25 H34 O13; 6.319 269.0558	**Deoxyloganin tetraacetate**	577.1738	6.319	Auto MS/MS	542.2048

MS Spectrum

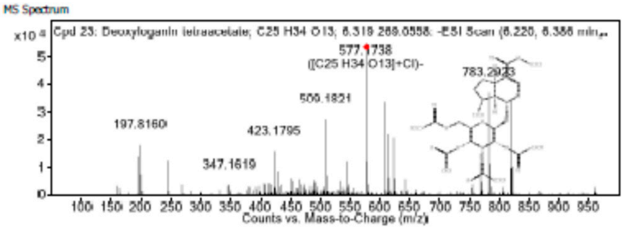

MS Zoomed Spectrum

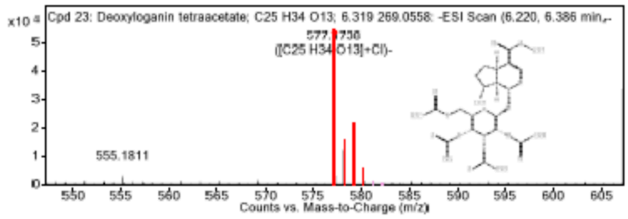

MS Spectrum Peak List

m/z	*Calc m/z*	Diff(ppm)	z	Abund	Formula	Ion
509.1821			1	27242.99		
577.1738	577.1693	-7.72	1	56260.04	C25 H34 O13	(M+Cl)-
578.1765	578.1728	-6.48	1	12249.46	C25 H34 O13	(M+Cl)-
579.1827	579.1678	-25.6	1	3028.44	C25 H34 O13	(M+Cl)-
580.1872	580.1707	-28.49	1	639.84	C25 H34 O13	(M+Cl)-
607.1844			1	34678.46		
613.1506			1	21984.45		
623.2158			1	21176.83		
783.2923			1	37841.18		
819.2693			1	34322		

MSMS Spectrum

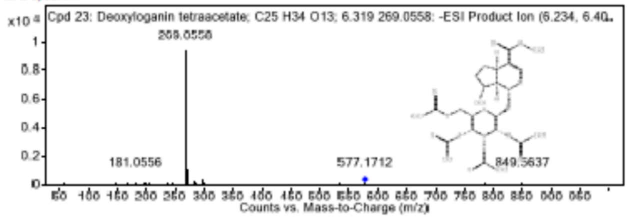

MS/MS Spectrum Peak List

m/z	z	Abund
59.0175		56.01
181.0556		73.92
245.102		55.55
268.0459		101.72
269.0558	1	9425.81
270.0588	1	1053.49
271.0625	1	86.81
284.0422		256.33
299.0657	1	282.83
300.0702	1	44.27

Compound Structure

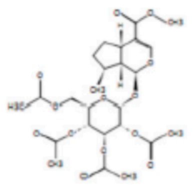

CMP-N-acetylneuraminic acid:

Compound Label	Name	*m/z*	RT	Algorithm	Mass
Cpd 24: CMP-N-acetylneuraminic acid; C20 H31 N4 O16 P; 6.329 269.0561	**CMP-N-acetylneuraminic acid**	613.1506	6.329	Auto MS/MS	614.1563

MS Spectrum

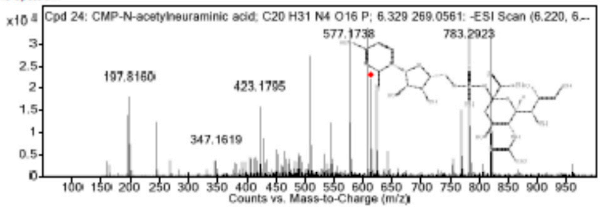

MS Zoomed Spectrum

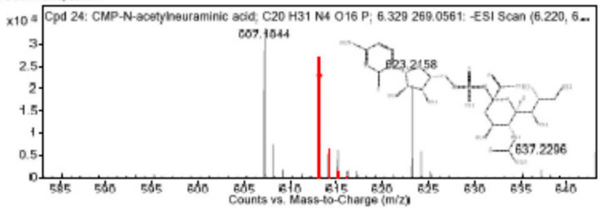

MS Spectrum Peak List

m/z	*Calc m/z*	Diff(ppm)	z	Abund	Formula	Ion
509.1821			1	27242.99		
577.1738			1	56260.04		
607.1844			1	34678.46		
613.1506	613.14	-17.24	1	21984.45	C20 H31 N4 O16 P	(M-H)-
614.153	614.143	-16.18	1	5462.68	C20 H31 N4 O16 P	(M-H)-
615.1494	615.1451	-7.05	1	6387.33	C20 H31 N4 O16 P	(M-H)-
616.1513	616.1476	-5.95	1	1518.66	C20 H31 N4 O16 P	(M-H)-
623.2158			1	21176.83		
783.2923			1	37841.18		
819.2693			1	34322		

MSMS Spectrum

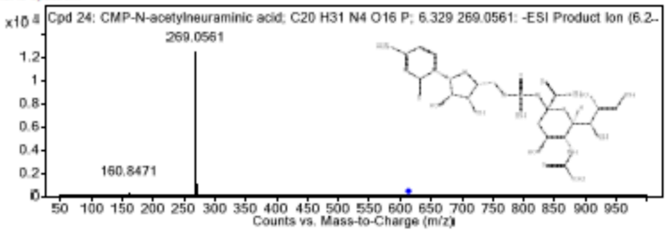

MS/MS Spectrum Peak List

m/z	z	Abund
59.0175		61.52
160.8471		173.9
162.8452		100.34
197.8157		67.41
268.0466		95.61
269.0561	1	12447.09
270.0596	1	1049.84
271.0601	1	126.4
299.0655		73.02
342.4462		50.12

Compound Structure

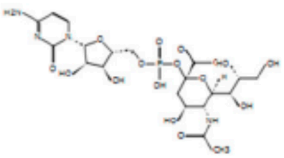

Carapin-8 (9)-Ene:

Compound Label	Name	*m/z*	RT	Algorithm	Mass
Cpd 38: CARAPIN-8 (9)-ENE; C27 H30 O7; 8.198 59.0174	**CARAPIN-8 (9)-ENE**	447.1813	8.198	Auto MS/MS	466.1996

MS Spectrum

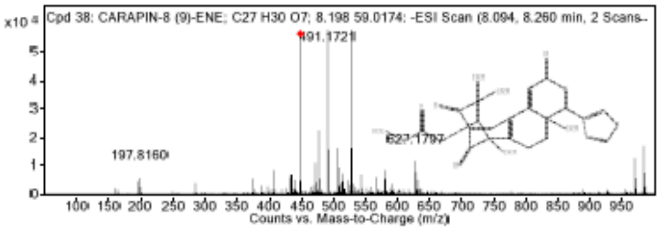

MS Zoomed Spectrum

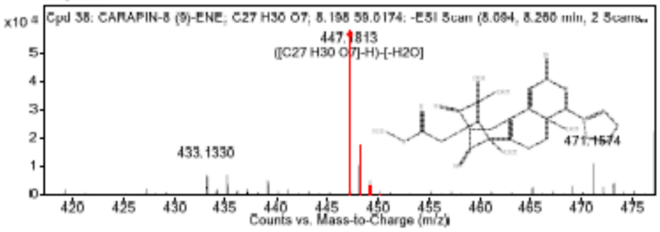

MS Spectrum Peak List

m/z	*Calc m/z*	Diff(ppm)	z	Abund	Formula	Ion
447.1813	447.1813	0.09	1	57765.43	C27 H30 O7	(M-H)-[-H2O]
448.1841	448.1847	1.27	1	10451.36	C27 H30 O7	(M-H)-[-H2O]
449.1939	449.1875	-14.12	1	4856.29	C27 H30 O7	(M-H)-[-H2O]
450.197	450.1903	-15.02	1	1100.7	C27 H30 O7	(M-H)-[-H2O]
477.192			1	23927.66		
491.1721			1	84565.56		
507.1357			1	16452.2		
527.1494			1	62940.32		
529.1506			1	16358.7		
983.3416			1	17349.83		

MSMS Spectrum

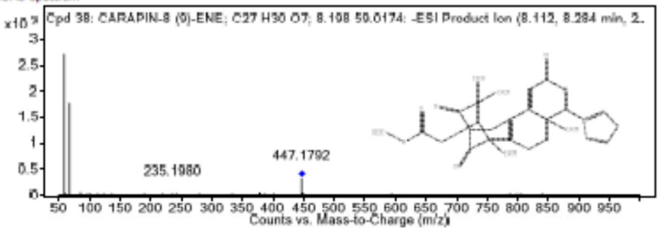

MS/MS Spectrum Peak List

m/z	z	Abund
59.0174	1	2712.94
60.0218	1	56.74
67.0223	1	1860.82
68.0259	1	165.41
85.0342		72.53
235.198		39.69
377.1738		50.03
378.1744		51.13
447.1792	1	336.17
448.1827	1	52.64

Compound Structure

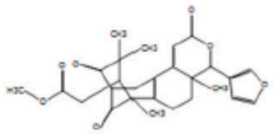

Selinidin:

Compound Label	Name	*m/z*	RT	Algorithm	Mass
Cpd 30: SELINIDIN; C19 H20 O5; 8.848 145.1000	**SELINIDIN**	311.127	8.848	Auto MS/MS	328.1303

MS Spectrum

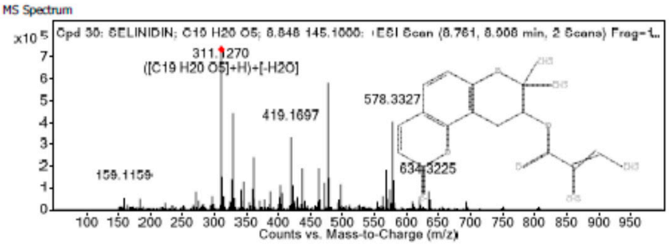

MS Zoomed Spectrum

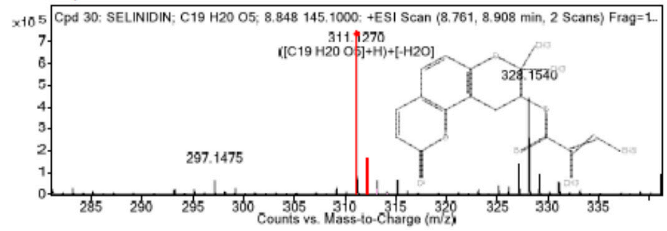

MS Spectrum Peak List

m/z	*Calc m/z*	Diff(ppm)	z	Abund	Formula	Ion
311.127	311.1278	2.65	1	789706.31	C19 H20 O5	(M+H)+[-H2O]
312.1305	312.1312	2.3	1	151321.51	C19 H20 O5	(M+H)+[-H2O]
328.154			1	459009.66		
361.1636			1	244102.12		
419.1697			1	334327.82		
436.1965			1	197296.62		
463.196			1	188665.86		
477.1754			1	586944.44		
568.2976			1	183620.93		
578.3327			1	404410.77		

MSMS Spectrum

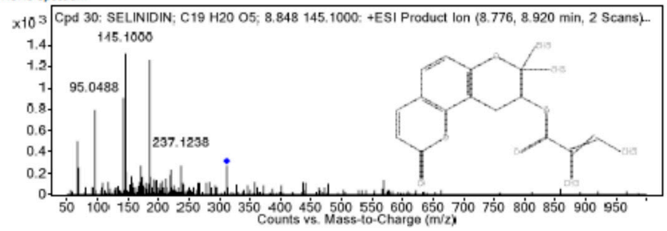

MS/MS Spectrum Peak List

m/z	z	Abund
67.0536		503.37
69.0324		254.95
95.0488		821.33
143.0849		910.85
145.1		1340.04
171.0794		281.46
185.0962	1	1277.22
221.1315		233.08
237.1238		282.02
311.1275		280.91

Compound Structure

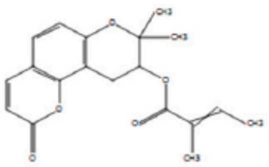

Harpagoside:

Compound Label	Name	*m/z*	RT	Algorithm	Mass
Cpd 35: Harpagoside; C24 H30 O11; 9.100 174.1479	**Harpagoside**	477.1748	9.1	Auto MS/MS	494.1782

MS Spectrum

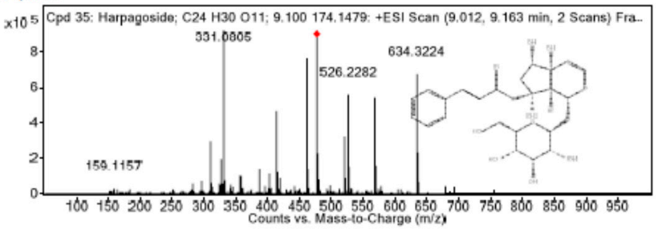

MS Zoomed Spectrum

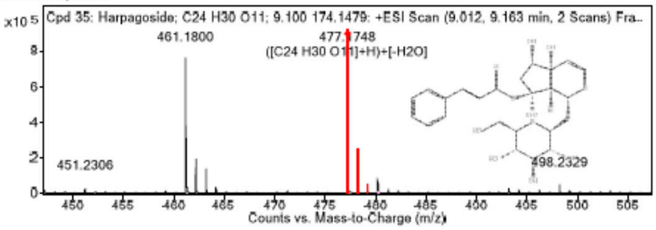

MS Spectrum Peak List

m/z	*Calc m/z*	Diff(ppm)	z	Abund	Formula	Ion
331.0805			1	2811009.9		
332.0837			1	497543.13		
414.1906			1	474010.73		
461.18			1	779009.93		
477.1748	477.1755	1.48	1	929190.9	C24 H30 O11	(M+H)+[-H2O]
478.1787	478.1789	0.56	1	230314.8	C24 H30 O11	(M+H)+[-H2O]
479.1824	479.1814	-2.09	1	50001.3	C24 H30 O11	(M+H)+[-H2O]
526.2282			1	563004.13		
568.2962			1	569283.16		
634.3224			1	685710.56		

MSMS Spectrum

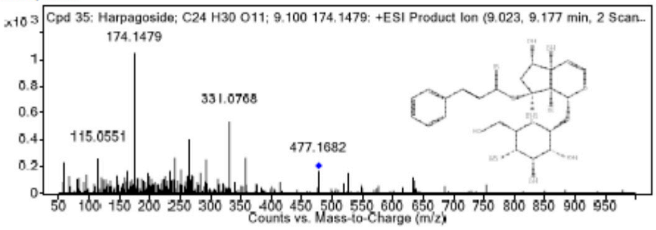

MS/MS Spectrum Peak List

m/z	Abund
59.0719	235.43
115.0551	257.02
174.1479	1061.1
241.1183	262.7
251.1037	176.52
265.1177	399.64
293.1119	255.33
331.0768	535.5
357.1297	270.75
477.1682	181.94

Compound Structure

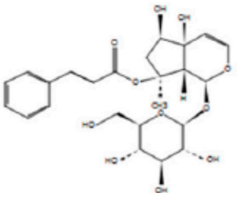

8. -Epiiridodial glucoside tetraacetate:

Compound Label	Name	*m/z*	RT	Algorithm	Mass
Cpd 36: 8-Epiiridodial glucoside tetraacetate; C24 H34 O11; 9.126 145.0639	**8-Epiiridodial glucoside tetraacetate**	521.2019	9.126	Auto MS/MS	498.2126

MS Spectrum

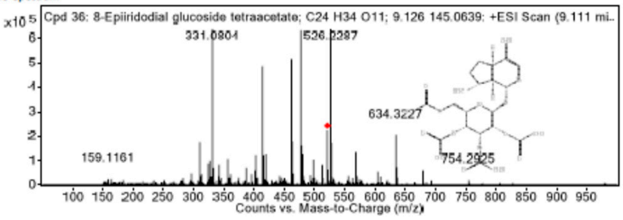

MS Zoomed Spectrum

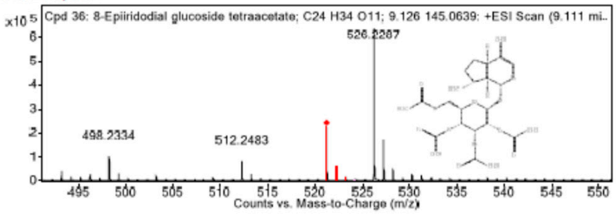

MS Spectrum Peak List

m/z	*Calc m/z*	Diff(ppm)	z	Abund	Formula	Ion
331.0804			1	2600404.07		
332.0842			1	418111.85		
414.1911			1	488627.2		
461.1809			1	517192.3		
477.1758			1	640045.54		
521.2019	521.1993	-4.96	1	225486.7	C24 H34 O11	(M+Na)+
522.2046	522.2027	-3.64	1	63736.68	C24 H34 O11	(M+Na)+
523.2094	523.2051	-8.22	1	15872.79	C24 H34 O11	(M+Na)+
526.2287			1	641512.35		
634.3227			1	206969.48		

MSMS Spectrum

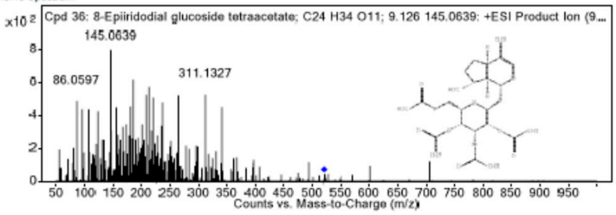

MS/MS Spectrum Peak List

m/z	Abund
86.0597	484.1
145.0639	836.35
181.1003	453.57
185.0946	617.03
209.091	556.22
213.0883	614.26
221.0938	506.63
237.1252	523.14
265.1207	557.93
311.1327	547.47

Compound Structure

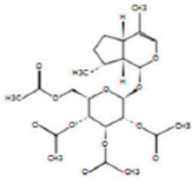

Larixol Acetate:

Compound Label	Name	*m/z*	RT	Algorithm	Mass
Cpd 42: LARIXOL ACETATE; C22 H36 O3; 9.262 299.0318	**LARIXOL ACETATE**	329.2458	9.262	Auto MS/MS	348.2635

MS Spectrum

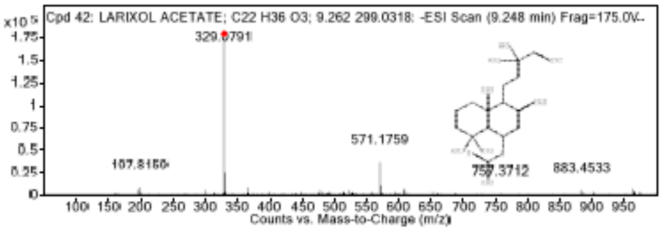

MS Zoomed Spectrum

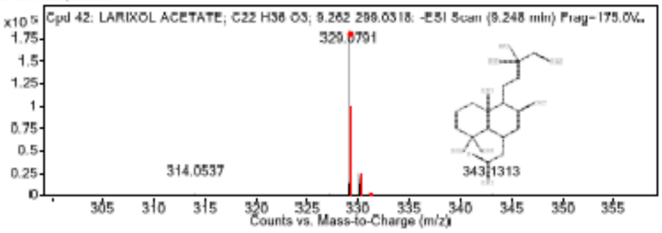

MS Spectrum Peak List

m/z	*Calc m/z*	Diff(ppm)	z	Abund	Formula	Ion
197.8159				7761.68		
329.0791			1	196528.67		
329.2458	329.2486	8.51	1	98972.11	C22 H36 O3	(M-H)-[-H2O]
330.0817			1	25291.59		
330.2481	330.252	11.96	1	14390.67	C22 H36 O3	(M-H)-[-H2O]
331.2499	331.2551	15.63	1	1659.68	C22 H36 O3	(M-H)-[-H2O]
365.2218			1	7731.56		
571.1759			1	37705.34		
572.1788			1	8328.68		
573.1753			1	10734.98		

MSMS Spectrum

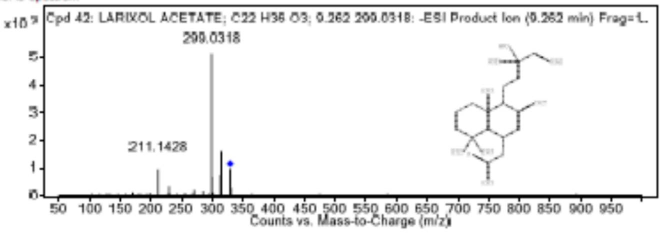

MS/MS Spectrum Peak List

m/z	z	Abund
211.1428		966.48
229.1548		353.03
299.0318	1	5170.82
300.0356	1	711.4
313.0479		725.21
314.0547	1	1648.58
315.0588	1	356.92
329.0802	1	1009.76
329.246	1	789
330.0811	1	284.99

Compound Structure

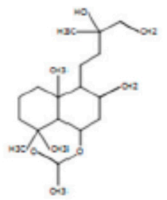

Valtratum:

Compound Label	Name	*m/z*	RT	Algorithm	Mass
Cpd 45: Valtratum; C22 H30 O8; 9.525 95.0488	Valtratum	405.1903	9.525	Auto MS/MS	422.1935

MS Spectrum

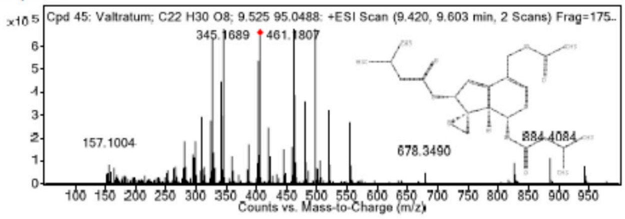

MS Zoomed Spectrum

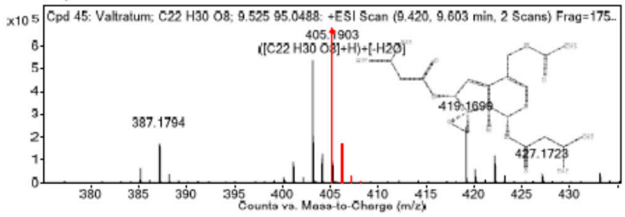

MS Spectrum Peak List

m/z	*Calc m/z*	Diff(ppm)	z	Abund	Formula	Ion
327.1583			1	672834.81		
345.1689			1	890710.19		
403.1749			1	538124.56		
405.1903	405.1908	1.3	1	678959.97	C22 H30 O8	(M+H)+[-H2O]
406.1936	406.1942	1.48	1	157740.69	C22 H30 O8	(M+H)+[-H2O]
407.1966	407.1967	0.27	1	27240.53	C22 H30 O8	(M+H)+[-H2O]
408.1977	408.1994	4.13	1	7391.83	C22 H30 O8	(M+H)+[-H2O]
461.1807			1	2178501.9		
462.184			1	548614.91		
496.2179			1	1377036.08		

MSMS Spectrum

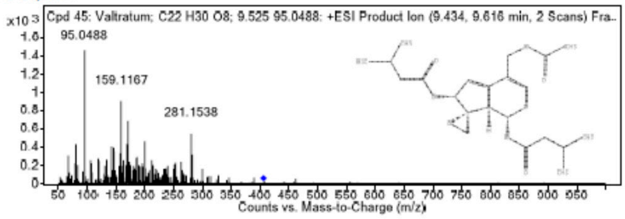

MS/MS Spectrum Peak List

m/z	Abund
81.0335	436.96
95.0488	1518.11
143.0834	404.19
145.0994	395.3
147.0823	362.48
159.1167	958.6
169.1003	437.59
171.1165	694.5
199.1124	491.66
281.1538	569.66

Compound Structure

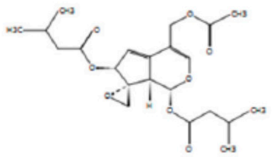

Triptonide:

Compound Label	Name	*m/z*	RT	Algorithm	Mass
Cpd 56: TRIPTONIDE; C20 H22 O6; 9.807 95.0490	**TRIPTONIDE**	341.1381	9.807	Auto MS/MS	358.1419

MS Spectrum

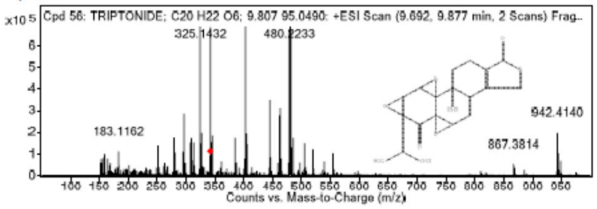

MS Zoomed Spectrum

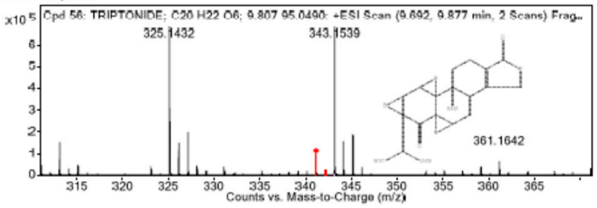

MS Spectrum Peak List

m/z	*Calc m/z*	Diff(ppm)	z	Abund	Formula	Ion
325.1432			1	747066.89		
341.1381	341.1384	0.68		94318.1	C20 H22 O6	(M+H)+[-H2O]
342.144	342.1417	-6.56	1	21318.7	C20 H22 O6	(M+H)+[-H2O]
343.1539			1	726491.5		
403.1751			1	846793.77		
445.1868			1	349534.67		
463.1965			1	308934.97		
478.2082			1	678502.57		
480.2233			1	2797858.27		
481.2269			1	688215.65		

MSMS Spectrum

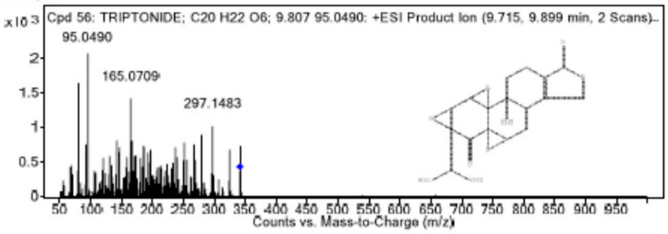

MS/MS Spectrum Peak List

m/z	Abund
81.0336	1653.34
93.0707	773.63
95.049	2201.01
143.0856	825.94
147.0799	769.82
165.0709	1496.25
167.0866	852.46
251.1444	789.52
279.1402	897.42
297.1483	1091.97

Compound Structure

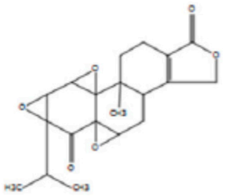

Koparin 2'-Methyl Ether:

Compound Label	Name	*m/z*	RT	Algorithm	Mass
Cpd 60: KOPARIN 2'-METHYL ETHER; C17 H14 O6; 10.036 282.0520	**KOPARIN 2'-METHYL ETHER**	315.0866	10.036	Auto MS/MS	314.0792

MS Spectrum

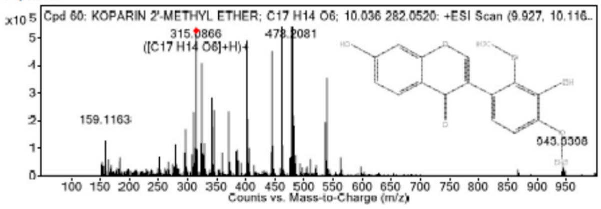

MS Zoomed Spectrum

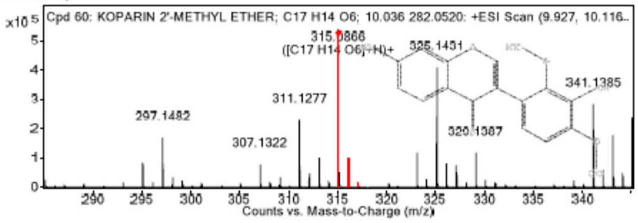

MS Spectrum Peak List

m/z	*Calc m/z*	Diff(ppm)	z	Abund	Formula	Ion
315.0866	315.0863	-0.75	1	537779.59	C17 H14 O6	(M+H)+
316.0893	316.0897	1.43	1	96611.49	C17 H14 O6	(M+H)+
317.0928	317.092	-2.4	1	14106.01	C17 H14 O6	(M+H)+
325.1431			1	407084.06		
401.1604			1	504304		
445.1859			1	451076.15		
461.1812			1	1366163.2		
478.2081			1	2090736.23		
479.2119			1	539578.05		
480.2225			1	953070.32		

MSMS Spectrum

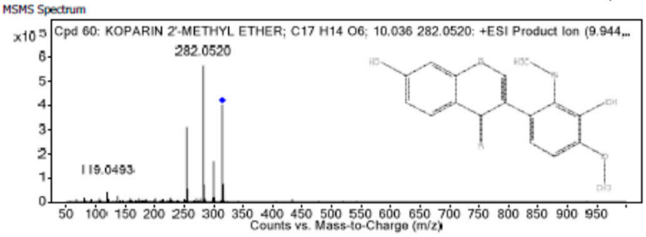

MS/MS Spectrum Peak List

m/z	z	Abund
119.0493		404.99
136.0146		223.8
226.0588		190.21
254.0571	1	3189.35
255.0589	1	547.64
282.052	1	5645.73
283.0565	1	739.42
300.0622	1	1702.22
315.0846	1	4050.72
316.0891	1	750.69

Compound Structure

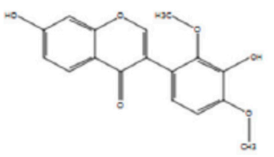

.

Dihydrosamidin:

Compound Label	Name	*m/z*	RT	Algorithm	Mass
Cpd 73: DIHYDROSAMIDIN; C21 H24 O7; 10.779 185.0955	**DIHYDROSAMIDIN**	371.1491	10.779	Auto MS/MS	388.1524

MS Spectrum

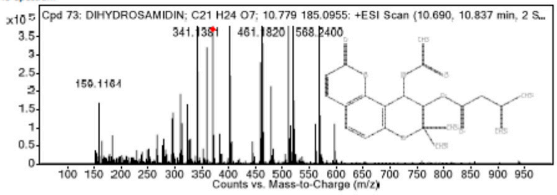

MS Zoomed Spectrum

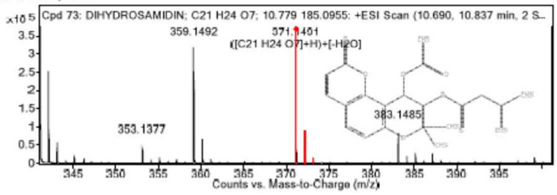

MS Spectrum Peak List

m/z	*Calc m/z*	Diff(ppm)	z	Abund	Formula	Ion
341.1381			1	1308695.76		
371.1491	371.1489	-0.57	1	378904.28	C21 H24 O7	(M+H)+[-H2O]
372.1518	372.1523	1.31	1	84138.65	C21 H24 O7	(M+H)+[-H2O]
373.1578	373.1549	-7.88	1	16187.92	C21 H24 O7	(M+H)+[-H2O]
401.1598			1	435222.97		
461.182			1	6357996.06		
462.1843			1	1783480.61		
510.234			1	1333914.38		
519.1869			1	644816.11		
568.24			1	454744.08		

MSMS Spectrum

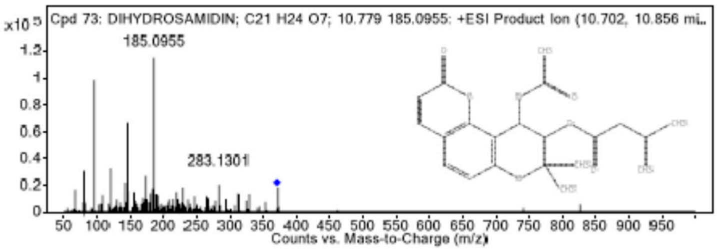

MS/MS Spectrum Peak List

m/z	Abund
81.0323	327.56
95.0485	1010.41
96.052	299.85
121.0639	321.98
143.0866	214.63
145.1	671.64
173.0921	273.1
185.0955	1149.29
283.1301	205.01
372.2412	179.59

Compound Structure

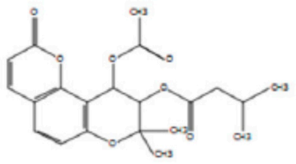

10. S,11R-Epoxy-punaglandin 4:

Compound Label	Name	m/z	RT	Algorithm	Mass
Cpd 83: 10S,11R-epoxy-punaglandin 4; C25 H35 Cl O9; 11.022 95.0485	**10S,11R-epoxy-punaglandin 4**	519.1681	11.022	Auto MS/MS	514.1895

MS Spectrum

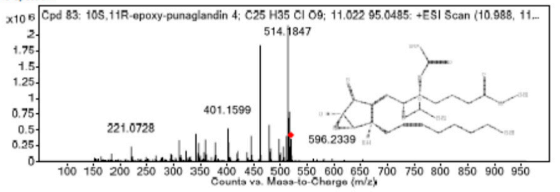

MS Zoomed Spectrum

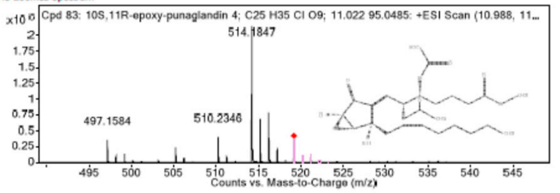

MS Spectrum Peak List

m/z	z	Abund
341.1383	1	439284.93
401.1599	1	530759.69
445.1865	1	398513.49
461.181	1	1854586.13
462.1851	1	462993.89
479.1479	1	576541.12
510.2346	1	402189.41
514.1847	1	2200054.95
515.1889	1	707963.68
516.1841	1	792510.98

MSMS Spectrum

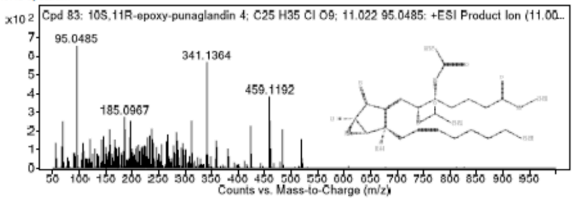

MS/MS Spectrum Peak List

m/z	Abund
69.0328	260.87
95.0485	672.96
157.1053	215.6
185.0967	282.22
197.099	254.23
311.128	255.91
341.1364	587.12
423.1443	236.75
459.1192	383.86
460.1248	261.04

Compound Structure

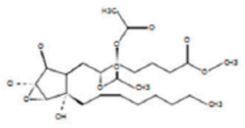

16alpha,17beta-Estriol 3-(beta-D-glucuronide):

Compound Label	Name	*m/z*	RT	Algorithm	Mass
Cpd 89: 16alpha,17beta-Estriol 3-(beta-D-glucuronide); C24 H32 O9; 11.279 159.1167	**16alpha,17beta-Estriol 3-(beta-D-glucuronide)**	447.2019	11.279	Auto MS/MS	464.2051

MS Spectrum

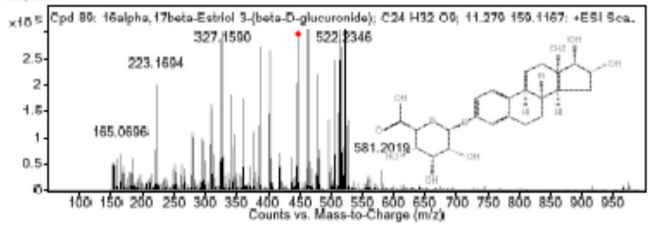

MS Zoomed Spectrum

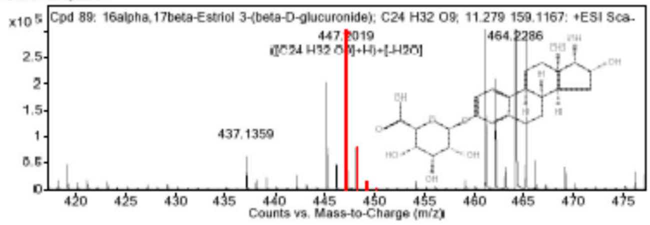

MS Spectrum Peak List

m/z	*Calc m/z*	Diff(ppm)	z	Abund	Formula	Ion
327.159			1	321014.75		
447.2019	447.2013	-1.26	1	304887	C24 H32 O9	(M+H)+[-H2O]
448.2048	448.2047	-0.17	1	80552.88	C24 H32 O9	(M+H)+[-H2O]
449.2074	449.2073	-0.12	1	15236.82	C24 H32 O9	(M+H)+[-H2O]
450.2134	450.21	-7.56	1	3266.52	C24 H32 O9	(M+H)+[-H2O]
461.1815			1	827278.5		
464.2286			1	1167843		
514.1846			1	809426.88		
522.2346			1	1992575.23		
523.2381			1	554158.08		

MSMS Spectrum

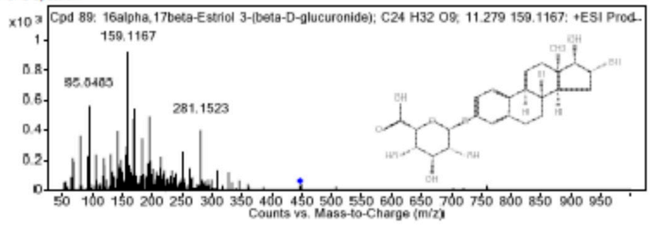

MS/MS Spectrum Peak List

m/z	Abund
81.0331	365.46
95.0483	567.74
143.0834	392.82
157.0998	284.01
159.1167	950.46
169.1014	476.18
171.116	565.32
183.1154	340.6
195.1151	490.53
281.1523	398.57

Compound Structure

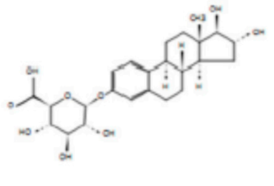

16. -Hydroxy-4-carboxyretinoic Acid:

Cpd 90: 16-Hydroxy-4-carboxyretinoic acid; C20 H24 O5; 11.280 95.0490	**16-Hydroxy-4-carboxyretinoic acid**	327.159	11.28	Auto MS/MS	344.1621

MS Spectrum

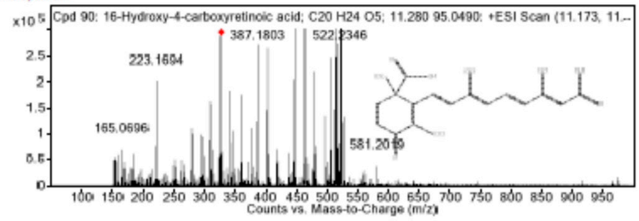

MS Zoomed Spectrum

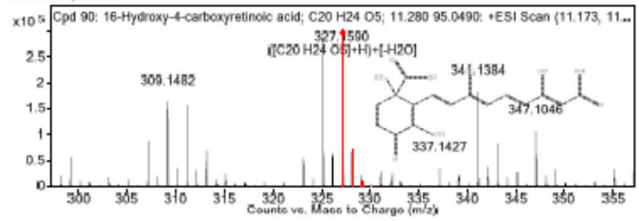

MS Spectrum Peak List

m/z	*Calc m/z*	Diff(ppm)	z	Abund	Formula	Ion
327.159	327.1591	0.36	1	321014.75	C20 H24 O5	(M+H)+[-H2O]
328.1617	328.1625	2.48	1	65482.73	C20 H24 O5	(M+H)+[-H2O]
329.1628	329.1652	7.46	1	13516.69	C20 H24 O5	(M+H)+[-H2O]
447.2019			1	304887		
461.1815			1	827278.5		
464.2286			1	1167843		
465.2321			1	302352.03		
514.1846			1	809426.88		
522.2346			1	1992575.23		
523.2381			1	554158.08		

MSMS Spectrum

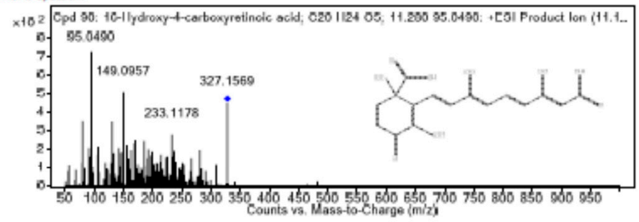

MS/MS Spectrum Peak List

m/z	Abund
81.0334	353.55
95.049	757.98
131.0836	349.69
149.0957	513.31
156.0918	224.93
169.1065	228.57
171.1176	248.61
185.0937	244.94
233.1178	285.36
327.1569	457.56

Compound Structure

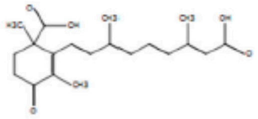

Isotectorigenin, 7- Methyl ether:

Compound Label	Name	m/z	RT	Algorithm	Mass
Cpd 95: ISOTECTORIGENIN, 7-METHYL ETHER; C18 H16 O6; 12.149 296.0673	**ISOTECTORIGENIN, 7-METHYL ETHER**	329.1011	12.149	Auto MS/MS	328.0939

MS Spectrum

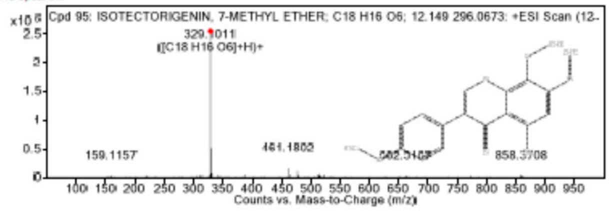

MS Zoomed Spectrum

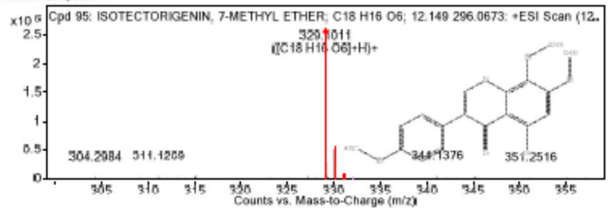

MS Spectrum Peak List

m/z	Calc m/z	Diff(ppm)	z	Abund	Formula	Ion
329.1011	329.102	2.56	1	2768248.3	C18 H16 O6	(M+H)+
330.1047	330.1054	2.07	1	519730.92	C18 H16 O6	(M+H)+
331.1067	331.1077	3.27	1	83234.42	C18 H16 O6	(M+H)+
387.1792			1	56027.27		
461.1802			1	164166.99		
464.2284			1	59146.58		
477.2113			1	120359.66		
510.2328			1	51435.17		
514.1836			1	71223.3		
522.2334			1	55925.88		

MSMS Spectrum

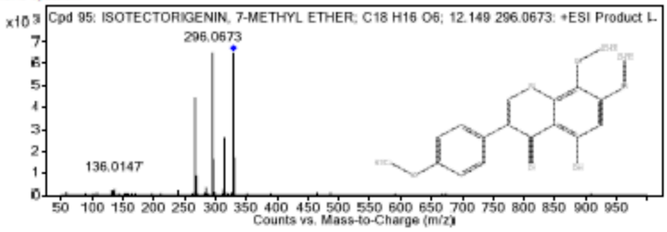

MS/MS Spectrum Peak List

m/z	z	Abund
268.0718	1	4580.15
269.0745	1	914
285.0713		353.32
296.0673	1	6787.21
297.0703	1	1651.59
313.0683		285.75
314.077	1	2725.31
315.0799	1	787.59
329.1007	1	6778.83
330.1044	1	1750.88

Compound Structure

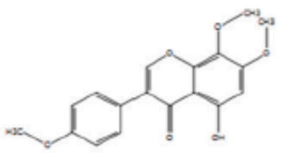

3. -hydroxy-3',4'- Dimethoxyflavone:

Compound Label	Name	m/z	RT	Algorithm	Mass
Cpd 97: 3-HYDROXY-3',4'-DIMETHOXYFLAVONE; C17 H14 O5; 13.274 299.0910	**3-HYDROXY-3',4'-DIMETHOXYFLAVONE**	299.0903	13.274	Auto MS/MS	298.0829

MS Spectrum

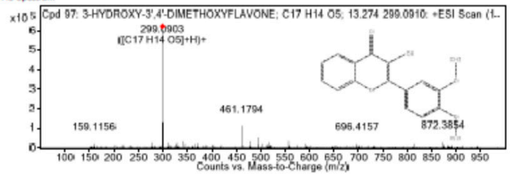

MS Zoomed Spectrum

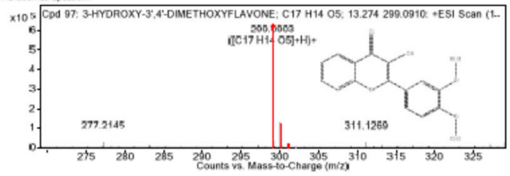

MS Spectrum Peak List

m/z	Calc m/z	Diff(ppm)	z	Abund	Formula	Ion
299.0903	299.0914	3.84	1	654240.11	C17 H14 O5	(M+H)+
300.0934	300.0948	4.71	1	130669.89	C17 H14 O5	(M+H)+
301.0962	301.0972	3.5	1	15875.14	C17 H14 O5	(M+H)+
341.1367			1	32267.02		
461.1794			1	112588.42		
462.1834			1	31031.02		
478.2569			1	34912.65		
494.2738			1	52750.95		
557.2377			1	36013.1		
872.3854			1	30900.82		

MSMS Spectrum

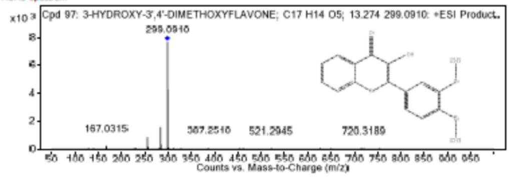

MS/MS Spectrum Peak List

m/z	z	Abund
167.0315		273.97
256.0725	1	870.12
257.0728	1	196.13
284.0674	1	1563.22
285.0684	1	328.6
299.091	1	7735.93
300.0951	1	1761.32
301.0977	1	128.19
387.251		111.75
720.3189		103.93

Compound Structure

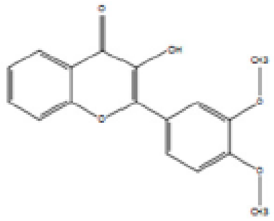

Khayanthone:

Compound Label	Name	*m/z*	RT	Algorithm	Mass
Cpd 98: KHAYANTHONE; C32 H42 O9; 18.427 533.2516	**KHAYANTHONE**	593.2746	18.427	Auto MS/MS	570.2854

MS Spectrum

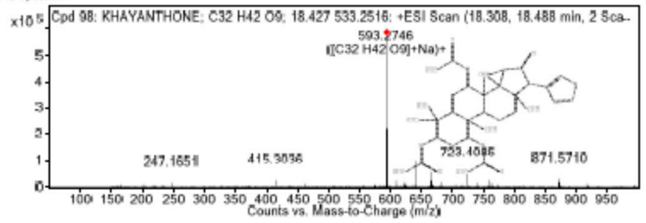

MS Zoomed Spectrum

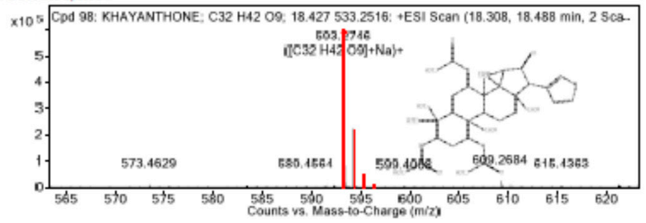

MS Spectrum Peak List

m/z	*Calc m/z*	Diff(ppm)	z	Abund	Formula	Ion
593.2746	593.2721	-4.26	1	601439.17	C32 H42 O9	(M+Na)+
594.2783	594.2755	-4.63	1	226937.09	C32 H42 O9	(M+Na)+
595.2803	595.2783	-3.31	1	46520.84	C32 H42 O9	(M+Na)+
596.2815	596.2811	-0.69	1	7216.08	C32 H42 O9	(M+Na)+
639.2796			1	104196.43		
640.2825			1	41519.54		
665.4038			1	59070.34		
723.4085			1	48748.31		
758.4454			1	28300.74		
871.571			1	29702.36		

MSMS Spectrum

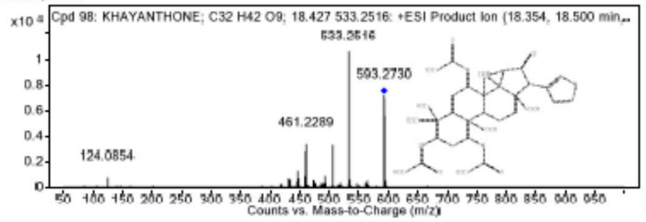

MS/MS Spectrum Peak List

m/z	z	Abund
447.2122	1	1282
460.2231		2797.96
461.2289	1	3491.84
492.2469		899.82
505.2209	1	3504.76
506.2228	1	1003.39
533.2516	1	10771.32
534.2554	1	3959.67
593.273	1	7176.39
594.2762	1	2953.29

Compound Structure

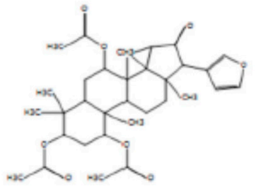

1-. dodecanoyl-sn-glycerol:

Compound Label	Name	*m/z*	RT	Algorithm	Mass
Cpd 48: 1-dodecanoyl-sn-glycerol; C15 H30 O4; 26.963 309.1849	**1-dodecanoyl-sn-glycerol**	309.1848	26.963	Auto MS/MS	274.215

MS Spectrum

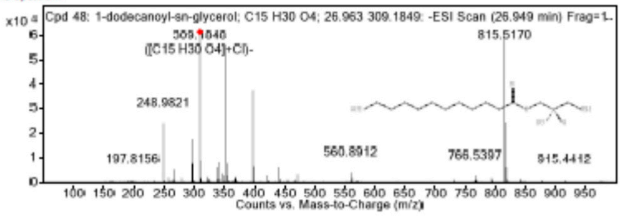

MS Zoomed Spectrum

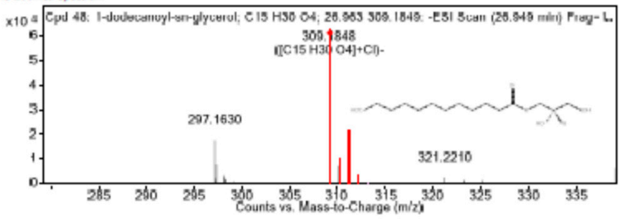

MS Spectrum Peak List

m/z	*Calc m/z*	Diff(ppm)	z	Abund	Formula	Ion
248.9821			1	25758.46		
297.163			1	17299.19		
309.1848	309.1838	-3.3	1	62633.89	C15 H30 O4	(M+Cl)-
310.1876	310.1872	-1.32	1	7244.13	C15 H30 O4	(M+Cl)-
311.1798	311.1814	5.2	1	8927.16	C15 H30 O4	(M+Cl)-
312.1821	312.1846	7.95	1	1528.8	C15 H30 O4	(M+Cl)-
353.2119			1	60227.08		
397.2388			1	38282.7		
815.517			1	61720.33		
816.5198			1	24202.39		

MSMS Spectrum

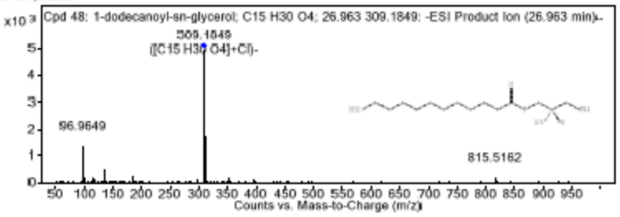

MS/MS Spectrum Peak List

m/z	*Calc m/z*	Diff (ppm)	z	Abund	Formula	Ion
96.9649				1318.81		
100.9657				153.43		
115.9253				141.71		
134.9009				487.28		
183.0187				209.47		
309.1849	309.1838	-3.66	1	4959.01	C15 H30 O4	(M+Cl)-
310.1868			1	716.28		
311.1795			1	1728.33		
353.2091				170.21		
815.5162				138.25		

Compound Structure

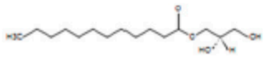

Supplement: Supplementary file 1 [file plants-09-01418-s001.pdf]
